# *Notes from the Field:* Increase in Eastern Equine Encephalitis Virus Activity — Vermont, 2023–2024

**DOI:** 10.15585/mmwr.mm7516a2

**Published:** 2026-04-30

**Authors:** Katherine M. Strelau, Emily Pareles, Patsy Kelso, Christine Matusevich, Patti Casey, Eliza Doncaster, Kaitlynn Levine, Carolyn V. Gould, J. Erin Staples, Kelly A. Fitzpatrick, Kristen L. Burkhalter, Cynthia Roxanne Connelly, Natalie A. Kwit

**Affiliations:** ^1^Epidemic Intelligence Service, CDC; ^2^Vermont Department of Health, Waterbury, Vermont; ^3^Vermont Agency of Agriculture, Food, and Markets; ^4^Division of Vector-borne Diseases, National Center for Emerging and Zoonotic Infectious Diseases, CDC.

**Summary**

**What is already known about this topic?**

Eastern equine encephalitis (EEE) is a rare, serious disease caused by the mosquitoborne EEE virus (EEEV). Approximately one third of human EEEV cases are fatal, and many survivors experience long-term neurologic sequelae.

**What is added by this report?**

During 2023–2024, increased EEEV activity was reported in northern Vermont; two human neuroinvasive disease cases (one fatal), three equine cases, and multiple EEEV-positive mosquito pools were reported.

**What are the implications for public health practice?**

CDC recommends that health departments use a One Health approach, including conducting EEEV surveillance in mosquitoes, susceptible domestic animals, and humans. Area-specific viral activity and risk levels should be communicated alongside mosquito bite prevention messaging to reduce the risk for infection.

Eastern equine encephalitis (EEE) virus (EEEV) is a mosquitoborne alphavirus maintained in an enzootic cycle with mosquitoes and birds. EEEV can be transmitted to humans and susceptible animals by mosquitoes that bite both mammals and birds. EEEV causes severe neuroinvasive disease in humans; although an EEE vaccine is available for horses, no human vaccine is currently licensed, and treatment is supportive. Approximately one third of human cases are fatal, and many survivors experience long-term neurologic sequelae (*1*). In the United States, a majority of EEE cases occur in states along the Atlantic Coast, Gulf Coast, and Great Lakes. EEEV was first detected in Vermont during a 2010 serosurvey of hunter-harvested deer and moose (*2*,*3*). After a 2011 outbreak of EEEV on a Vermont emu farm (*4*), statewide mosquito surveillance for EEEV was implemented in 2012. During 2012–2022, two human and four animal EEE cases were reported to the Vermont Department of Health (VDH). During 2023–2024, EEEV activity in mammals and mosquitoes increased, prompting targeted outreach in affected areas. This report describes EEEV activity in Vermont during 2023–2024 based on human and equine cases and mosquito surveillance data.

## Investigations and Outcomes

### Data Source

Each year during June–October, the Vermont Agency of Agriculture, Food, and Markets (VAAFM) traps, identifies, and pools* mosquitoes collected from approximately 100 sites throughout the state. Weekly EEEV testing of mosquito pools is conducted using reverse transcription–polymerase chain reaction testing at VDH and CDC laboratories. Laboratory EEEV detections and suspected human and animal cases are reportable to and investigated by VDH in coordination with VAAFM (for animal cases). This investigation was reviewed by CDC, deemed not research, and conducted consistent with applicable federal law and CDC policy.^†^

### EEEV Mosquito Detections

During 2012–2015, EEEV was detected in 42 mosquito pools (median = nine positive pools per year); no virus was detected in pools during 2016–2022 (Figure). In 2023, EEEV was detected in 14 mosquito pools from three towns, and in 2024, in 86 pools from 16 towns in northern and western Vermont. (In Vermont, a town is the primary subcounty administrative unit.) Among these 100 mosquito pools, the most common species to test positive for EEEV were *Culiseta melanura*, the primary enzootic vector (64); *Coquillettidia perturbans*, the primary vector that feeds on humans, horses, and birds (a bridge vector) (nine); and *Culex pipiens-restuans*, another bridge vector (six).

**FIGURE:**
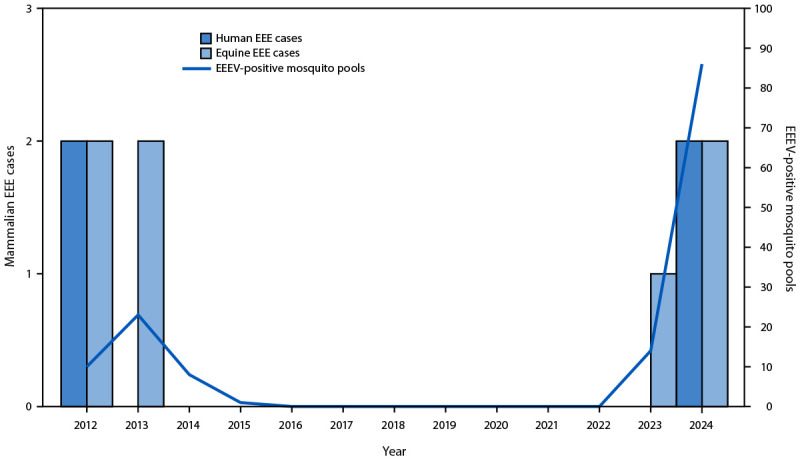
Number of eastern equine encephalitis cases in humans and horses and eastern equine encephalitis virus detections in mosquito pools— Vermont, 2012–2024 **Abbreviations:** EEE = eastern equine encephalitis; EEEV = eastern equine encephalitis virus.

### EEE Cases in Humans and Horses

**Human cases.** In 2012, the first two human EEE cases were reported in Vermont. No cases were reported again until 2024, when two men from the same northwestern county contracted neuroinvasive EEE; one was identified in July before any mosquito detections and survived, and the second patient died from his illness in early September.

**Equine cases.** During 2012–2013, VDH investigated four equine EEE cases; three horses died. None had a history of travel or veterinarian-administered EEE vaccination. No equine cases were reported during 2014–2022. During 2023 and 2024, three equine EEE cases were reported from northern Vermont; none had recent travel or documentation of receipt of recommended EEE vaccination.^§^ None of the horses survived. 

### Public Health Response

In response to increased EEEV detections in mosquitoes and mammals, VDH and VAAFM continued surveillance coordination, planned for potential vector control activities, and increased the volume of EEE risk and prevention communications. This response included preparing permits, contracts, and outreach plans in the event that aerial adulticiding^¶^ was needed, a municipal health briefing, health advisories, press releases, social media posts, weekly town health officer notifications, and multilingual flyers posted in areas where infected mosquitoes had been found. Wearing protective clothing and Environmental Protection Agency–approved repellents and limiting outdoor events and activity when mosquitoes are biting during dawn and dusk were recommended. Equine EEE vaccination was also recommended; however, because horses are dead-end hosts (i.e., they do not produce sufficient virus to transmit it back to mosquitoes), vaccination does not disrupt the transmission cycle or reduce the human public health risk.

## Preliminary Conclusions and Actions

Since the 1930s, EEE outbreaks in the northeastern United States have become more frequent and have expanded northward, with a record-breaking surge in 2019 (*5*). The reasons for this increase are unknown but are likely tied to climate and landscape changes, human behavior, increases in mosquito and bird populations, and evolving diagnostic and surveillance practices. To prevent and control EEEV transmission, CDC recommends that public health departments use a multispecies, One Health approach to surveillance, implement integrated vector management, and engage in risk mitigation communication strategies. Clinicians and veterinarians should consider EEE in human and equine patients with acute febrile or neurologic illness, especially during summer and fall, and ensure annual vaccination of horses before mosquito season in regions with endemic EEEV.

## Footnotes

* A mosquito pool is a group of one–50 mosquitoes of the same species, collected at the same trap location, on the same date.

† 45 C.F.R. part 46.102(l)(2), 21 C.F.R. part 56; 42 U.S.C. Sect. 241(d); 5 U.S.C. Sect. 552a; 44 U.S.C. Sect. 3501 et seq.

§ Vaccination for EEE is considered a core vaccine for all horses in the United States per the American Association of Equine Practitioners, and administration is recommended annually before the beginning of mosquito season, with consideration for 6-month intervals in areas that pose a high risk for infection.

¶ The application of insecticides to control adult mosquito populations and prevent the spread of mosquitoborne diseases.
